# Supplementary material for: The modular biochemical reaction network structure of cellular translation
Source: NPJ Syst Biol Appl. 2023 Oct 26;9:52. doi: 10.1038/s41540-023-00315-3 (PMC10603163; doi:10.1038/s41540-023-00315-3)
Supplement: Supplementary file 1 — Supplemental Material [file 41540_2023_315_MOESM1_ESM.pdf]

Supplementary Material

# The modular biochemical reaction network structure of cellular translation

Bruno Cuevas-Zuviría [1,2] , Evrim Fer [1,3], Zachary R. Adam [1,4] and Betül Kaçar [1,*]

[1] Department of Bacteriology, University of Wisconsin-Madison, Madison, WI, USA
[2] Centro de Biotecnología y Genómica de Plantas, Universidad Politécnica de Madrid (UPM), Instituto Nacional de Investigación y Tecnología Agraria y Alimentaria (INIA-CSIC), Madrid, Spain
[3] Microbiology Doctoral Training Program, University of Wisconsin-Madison, Madison, WI, USA
[4] Department of Geosciences, University of Wisconsin-Madison, Madison, WI, USA

*To whom correspondence should be addressed: bkacar@wisc.edu

## Supplementary Tables

Supplementary Table 1: A Description of all supplementary data files.

| File | Description | File format |
|---|---|---|
| **Supplementary Data 1** | Literature synthesis of translation reactions. | XLSX |
| **Supplementary Data 2** | Curated list of reactions (sheet 2) and compounds (sheet 1) involved in the translation reaction. | XLSX |
| **Supplementary Data 3** | Translation network, represented as a bipartite graph in a computer-readable format. It includes the reactions for elongation, termination, initiation, biosynthesis, stress-response, and cofactor-regeneration. | GML (Gephi, NetworkX) |
| **Supplementary Data 4** | Metabolic reactions, obtained from Fesit et al.[80], employed in this article. | XLSX |
| **Supplementary Data 5** | Metabolic network, represented as a bipartite graph in a computer-readable format. It includes the metabolic reactions and the molecules they are involved in. | GML |
| **Supplementary Data 6** | Biosynthesis network, represented as a bipartite graph in a computer-readable format. It includes the biosynthetic pathways to generate multimeric enzymes and the metabolic reactions they are involved in. | GML |
| **Supplementary Data 7** | Full network, including biosynthesis, metabolism, and translation, represented as a bipartite graph in a computer-readable format. It is a combined representation of the information presented at the Supplementary Data sets 3, 5 and 6. | GML |

# Supplementary Figures

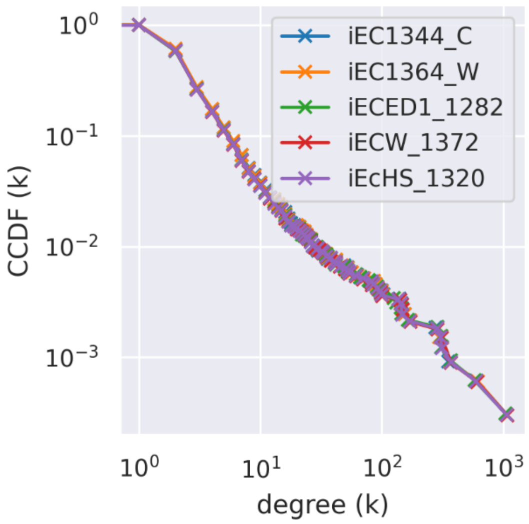


**Supplementary Figure 1**. Degree complementary cumulative distribution function (CCDF) of the compounds of five different strains.

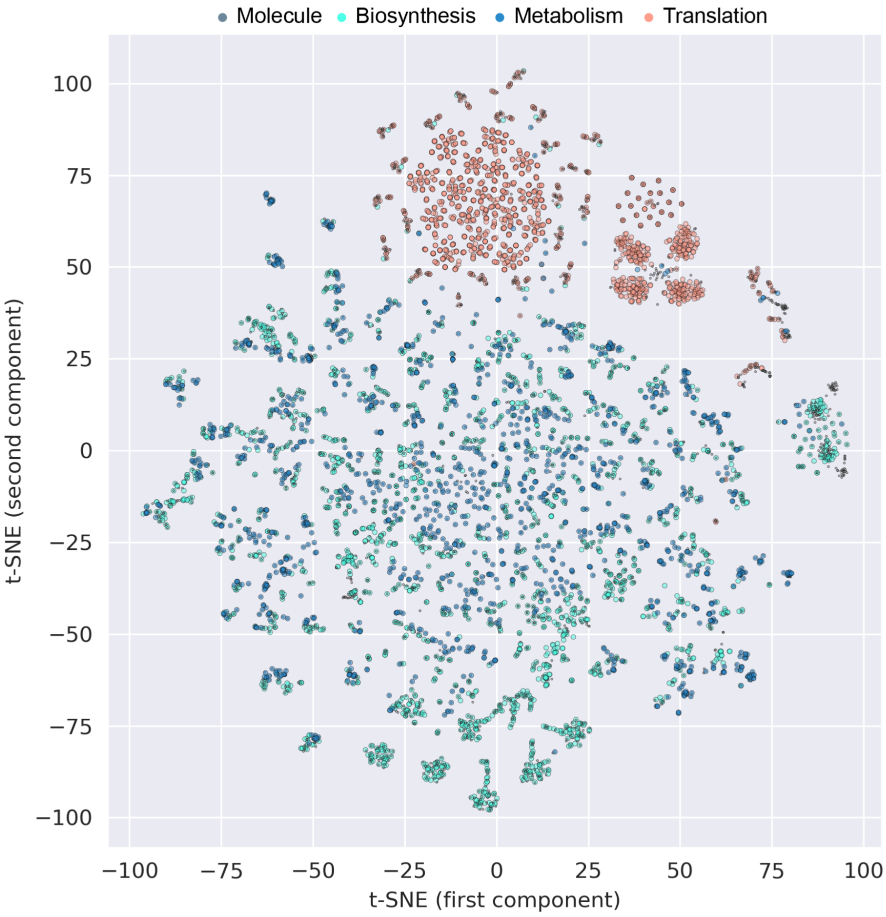


**Supplementary Figure 2**. Scatter plot of the t-distributed stochastic neighbor embedding (t-SNE) dimensional reduction of node2vec embeddings of reactions from the complete integrated network. Reactions within the different layers are color coded with all chemical compounds depicted in gray and layer reactions from Translation in orange, Biosynthesis in green and Metabolism in blue. Reactions from the translation and metabolism layers group distinctly from one another with little overlap. Reactions from the biosynthesis layer do not group together in any distinct way, but they broadly plot in the same areas that contain metabolic reactions.

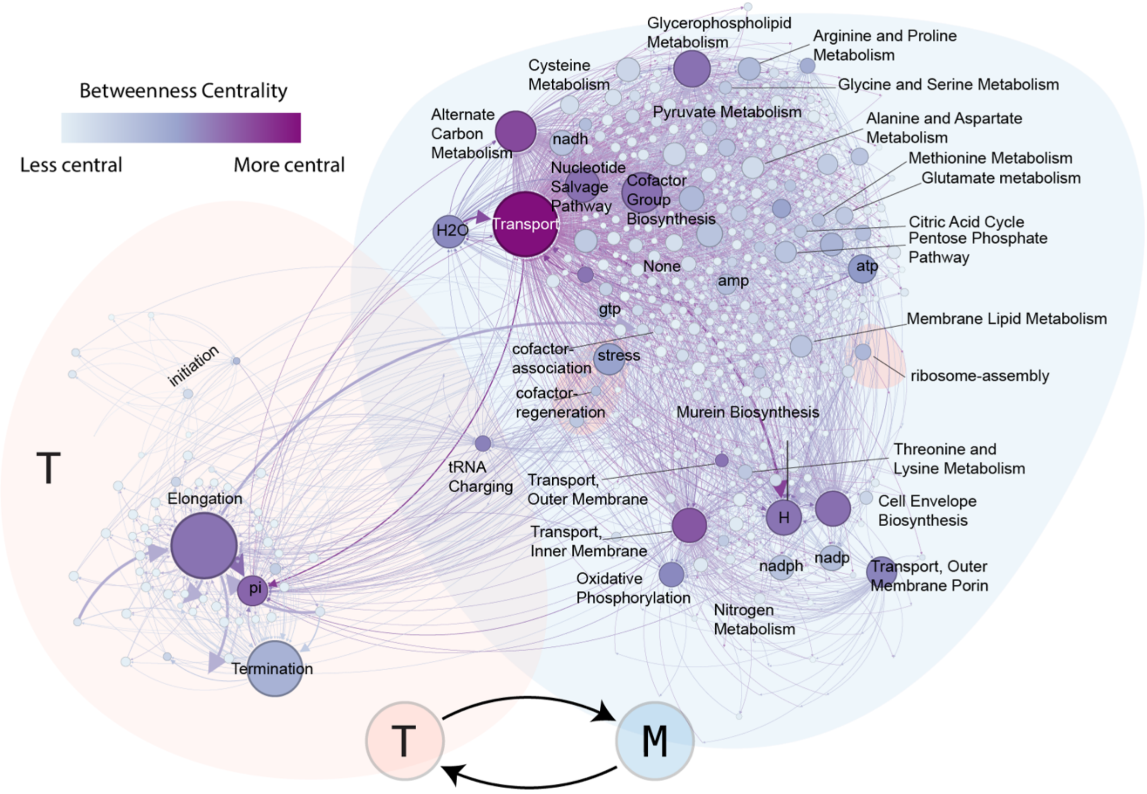


**Supplementary Figure 3**. Simplified view of the integrated network depicting the connections between translation and metabolism modules. A module is a collection of reactions and compounds that only connect to other reactions and compounds within the same assignment category. Compounds that occur in more than one module are conserved (*i.e.*, not reduced to a module). Module categories for translation were assigned during layer construction using labels as shown in Figure 2B, while those for metabolism were taken without modification or alteration from the source document from Feist et al[80]. Modules are coded from white (less central) to purple (more central) using the betweenness centrality metric; the more visited a node is in a path between two random nodes, the higher its betweenness-centrality.

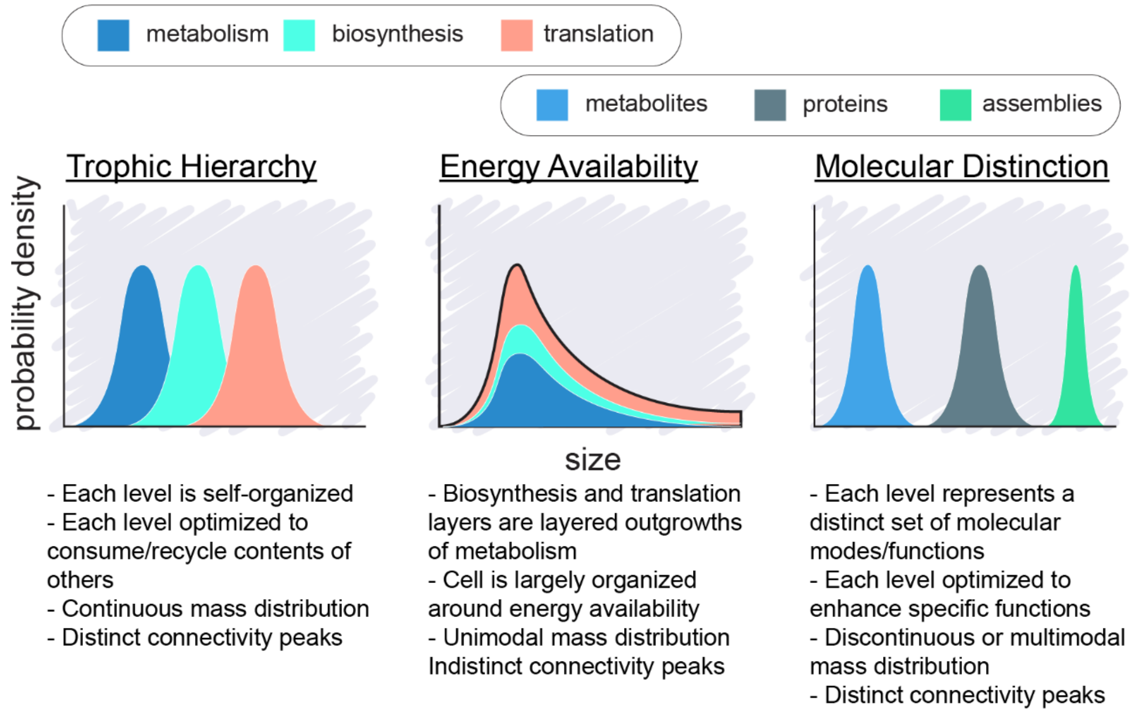


**Supplementary Figure 4**. Depiction of three different expected patterns of compound size distributions (top panels) and their respective associated characteristics (bottom panels).
